# Effect of Adding Cerium on Microstructure and Morphology of Ce-Based Inclusions Formed in Low-Carbon Steel

**DOI:** 10.1038/srep46503

**Published:** 2017-05-09

**Authors:** Z. Adabavazeh, W. S. Hwang, Y. H. Su

**Affiliations:** 1 Department of Materials Science and Engineering, National Cheng Kung University, No. 1 Tainan, 701, Taiwan; 2 Iron and Steel Research and Development Department, China Steel Corporation, Kaohsiung 812, Taiwan

## Abstract

Intra-granular Acicular Ferrite (IAF), as one of the most well-known desirable microstructure of ferrite with a chaotic crystallographic orientation, can not only refine the microstructure and retard the propagation of cleavage crack but also provide excellent combination of strength and toughness in steel. The effect of adding cerium on microstructure and controlling proper cerium-based inclusions in order to improve properties in low-carbon commercial steel (SS400) were investigated. The type of inclusions can be controlled by changing S/O ratio and Ce content. Without Ce modification, MnS is a dominate inclusion. After adding Ce, the stable inclusion phases change from AlCeO_3_ to Ce_2_O_2_S. The optimum amount of cerium, 0.0235 wt.%, lead in proper grain refinement and formation of cerium oxide, oxy-sulfide and sulfide inclusions. Having a high amount of cerium results in increasing the number of inclusions significantly as a result it cannot be effective enough and the inclusions will act like barriers for others. It is found that the inclusions with a size of about 4∼7 μm can serve as heterogeneous nucleation sites for AF formation. Thermodynamic calculations have been applied to predict the inclusion formation in this molten steel as well, which show a good agreement with experimental one.

Recently, the issues concerning non-metallic inclusions in steels have become one of the leading subjects of research in the field of metallurgy due to its important effect on the quality of steel. Before, non-metallic inclusions were treated as a detriment but indispensable product of deoxidation and desulphurization of steel, which should be removed or modified as much as possible so it did not decrease mechanical properties. Many researchers have investigated on improving the behavior of non-metallic inclusions during solidification of steel. It was found that depending on the chemical composition and their size, they can have different impact on properties of steel, starting from strongly negative to almost conditioning obtaining desired mechanical properties^1^,^2^,^3^,^4^.

Lots of studies have indicated that the inclusions, such as Ti, Al, and Zr oxides, Ti, Nb, and V carbonitrides, would contribute to the IAF nucleation, and the optimal heterogeneous nucleus was Ti_2_O_3_^5^,^6^,^7^,^8^,^9^. Many researchers reported that anisotropic microstructure and elongation of deformable MnS inclusions often happen as a result of the metal forming processes such as rolling and forging. Although the adding sulfur improves the machinability of steel, anisotropy in mechanical and fatigue properties would occur due to the presence of deformable MnS inclusions. In order to modify MnS inclusions, different methods are used by adding of Ca, REM (Rare-Earth-Metals) or Zr in the melt. The modification of non-metallic inclusions by means of Ca-treatment of liquid steels are often limited by the low and unstable yield of the added Ca, due to the high vaporization and low solubility of Ca in the liquid steel. Therefore, steelmaking companies prefer to use some other elements with higher vaporization temperature in the melt such as REM and Zr to modify sulfide inclusions^10^.

The studies about the effect of rare earth (RE) elements on the welding microstructures and properties indicated that the RE could react with O and S with the result of forming the high-melting point RE_x_O_y_, RE_x_S_y_ and RE_x_O_y_S_z_^5^. The key factors for the nucleation of intergranular bainite or acicular ferrite are the control of austenite grain size as well as the adjustment of the nature and size of non-metallic inclusions, which are both considered as favorable phases for mechanical properties at room temperature. The Gibbs’ free energies of these compounds at high temperatures are so low that REM elements can combine readily with oxygen and sulfur when added to liquid steel^11^,^12^. RE elements, with a strong affinity to oxygen and sulfur, were widely applied to spherodizing inclusions (such as MnS) to avoid the anisotropy of mechanical properties in final rolling products.

Recently it is reported that Ce_2_O_3_ with a low misfit value with ferrite can act as the nucleation sites for IAF under fast cooling rate. However, the inclusion characteristic and microstructure of furnace-cooled RE containing sample and inclusion formation evolution have not been discussed yet. Zhang *et al*.^13^ investigated the ability of Mg-based inclusions to induce AF nucleation in SS400 steel, which was Mg-treated using a commercial process. Their results showed that the magnesium-based complex inclusions could act as nucleation sites of AF. Inclusions with a size of about 5 *μ*m can serve as heterogeneous nucleation sites for AF.

Anmark *et al*.^10^ reviewed and summarized the effect of different non-metallic inclusions on the machinability of various steels. He mentioned that the magnitude of the effect of non-metallic inclusions on the improvement of the machinability of steel matrix, depends on the difference in the thermal expansion coefficients, α, between the steel matrix and the non-metallic inclusions. In the case that the non-metallic inclusions have different compositions and α coefficient than that of the steel matrix, the steel machinability can be affected. However, the value of the α coefficients can be different based on the contents of carbon and alloying elements. He concluded that the effect of the oxides and sulfides of REM and Zr on improving of machinability will be higher than the effect of MnS inclusions, which can be explained by the significantly lower magnitude of the difference between the values of α_*MnS*_ and α_*steel*_ for the stainless and high alloyed steels.

Different researchers worked on different aspects of adding REM in steels such as mechanisms of inclusion evolution, REM effects on the mechanical properties and impact toughness, and *in situ* observation of the evolution of IAF^4^,^5^,^11^,^14^. Deng *et al*.^4^ proposed a possible inclusion evolution mechanism based on calculated results using both calculations and experiments. Bin *et al*.^5^ worked on the *in situ* observation of the evolution of intragranular acicular ferrite at Ce-containing inclusions in 16Mn Steel. He also reported that the optimum content of Ce in 16Mn steel is around 0.02 wt%. Although different researchers have been investigated the effects of REM in steels, a comprehensive research in this field for low carbon steel, is still needed. The present study is designed to investigate the effect of cerium addition for grain refinement of SS400 steel comprehensively. The proper amount of Ce and controlling the type of inclusions have been discussed in details in order to find a reasonable relationship for industrial applications.

## Experimental Procedure

Commercial SS400 steel was melted at 1873 K in a vacuum induction furnace (100 kHz). Once the alloy was melted in a furnace under argon gas atmosphere, the melt was deoxidized with adding different amount of cerium powder wrapped in pure aluminum foil (99.99%). In order to control the type of Inclusions, S/O ratio has been changed. A wide range of samples are prepared, then the furnace power was turned off and crucible with the melt for the sample was slowly cooled down in the furnace, finally quenched with water. The chemical composition of as-cast samples is analyzed and presented in Table 1.

The amount of cerium is analyzed by ICP-AES method. In order to clarify the inclusions in steel samples, 1 × 1 × 1 cm^3^ cubic samples were cut from sample steel, then ground and polished using 3 and 1 μm diamond compound. A wide range of characterization methods are used in this research including Laser Scanning Confocal Microscopy (LSCM), ASPEX Explorer SEM/EDS, Scanning Electron Microscopy (SEM-EDS) and Optical Microscope (OM). The *in situ* observation of microstructure transformation was carried out using LSCM on cylindrical specimens, 8 mm in diameter and 10 mm in length. The sample for inclusion analysis was machined and then ground and polished using diamond compound. Metallographic observations were carried out on the specimens subjected to casted state. The statistic of grain size and inclusions were examined by using image statistical analysis. The types of inclusions and their morphology were extensively analyzed by SEM equipped with energy dispersive X-ray spectroscopy (EDX). In the present study, using ASPEX, the size distribution, composition, number and morphology of inclusions are automatically obtained for each sample. The total area examined for this test was 89.653 mm^2^. For observation of the microstructures, OM is employed for the samples etched for 1–2 mins using 3% Nital.

## Results and Discussion

### Thermodynamic analysis of rare earth inclusions formed in SS400

Interaction parameters according to Wagner^15^ and Lupis^16^ and Elliott have been very successfully used in the study of deoxidation reactions of steel for many years^17^,^18^. The interaction parameters bear significant correlation with properties that have physical meaning such as heat of formation of the corresponding oxides and atomic number of the deoxidants. These correlations not only help support the soundness of the formalism but also provide an interesting and useful way of checking the consistency of data presented in this formalism, as shown in A. Costa e Silva’s work^17^. He investigated the interaction parameters of oxygen and deoxidants in liquid iron.

Many researchers were in search of a mathematical way of handling the behavior of solutes in dilute solutions and were aware of the limitations this approach would have for less diluted solutions. This lead to the formalism of interaction coefficients for dilute solutions, widely used today^17^. In the addition of rare earth elements to the molten steel, there is a strong affinity among Ce and O and S. As a result, the thermodynamic calculation can be applied to derive the thermodynamic equations of inclusion formation in this steel. It is reported in the literature^14^ that the effect of rare earth is optimal when w (RE)/(w [O] + w [S]) = 3.9. Henrian activity coefficients and Henrian activities (1 wt% standard state) of O, S, Ce and Al in liquid steel can be predicted by interaction coefficients with Wagner’s model. In order to control the inclusions in practice, the thermodynamic analysis for the formation of the inclusions is performed. The activity coefficient of each element and activity in liquid steel is calculated by Eqs. (1) and (2):


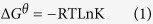



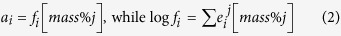


Where 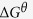 denotes the Gibbs free energy of reaction with the unit of J.mol;^−1^ R is the gas constant with the unit of J.mol^−1^.K;^−1^ T is temperature with unit of Kelvin; K is the equilibrium constant (without unit); a_i_ is the activity of element i; f_i_ is the Henrian activity coefficient of component i in dilute solution; 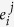 is the first-order interaction parameters i and j; w[i], w[j] are the mass percentage of elements i and j, respectively.

Standard Gibbs free energy change for formation of Ce_2_O_3_ and Ce_2_O_2_S in liquid steel is expressed respectively by Eq. (3) and (4)^14^.


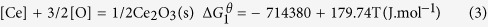



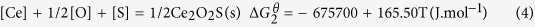


In addition to cerium oxides and oxy-sulfides, cerium sulfide also can be found in this type of steel. The formation of different type of cerium sulfides may be controlled by following equations^19^.


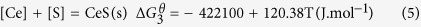



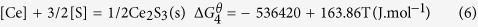



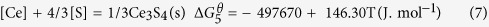


According to Eq. (8) and (9), the adding rare earth elements can react with the existing Al_2_O_3_ to form CeAlO_3_ or the rare earth elements directly react with oxygen and aluminum to form CeAlO_3_.


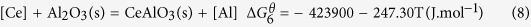



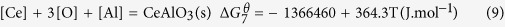


The activity coefficient of each element and activity in liquid steel is calculated by Eqs. (1) and (2). The basic chemical compositions are shown as No. 4 in Table 1. Table 2 shows the interaction coefficient 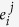
of various elements in liquid steel at 1873 K. The corresponding interaction coefficients of O, S, Ce and Al, at 1873K are used from previous researchers^19^,^20^. On the basis of the data, Henrian activity coefficients of O, S, Ce and Al in liquid steel containing 0.0235 wt% rare earth elements at 1873 K are calculated as 0.111, 0.926, 0.020 and 1.006, respectively. According to them Henrian activities are performed as well. The value of Raoultian activities of Ce_2_O_3_, Ce_2_O_2_S, and CeAlO_3_ is assumed to be unity^19^,^21^.

**Figure 1:**
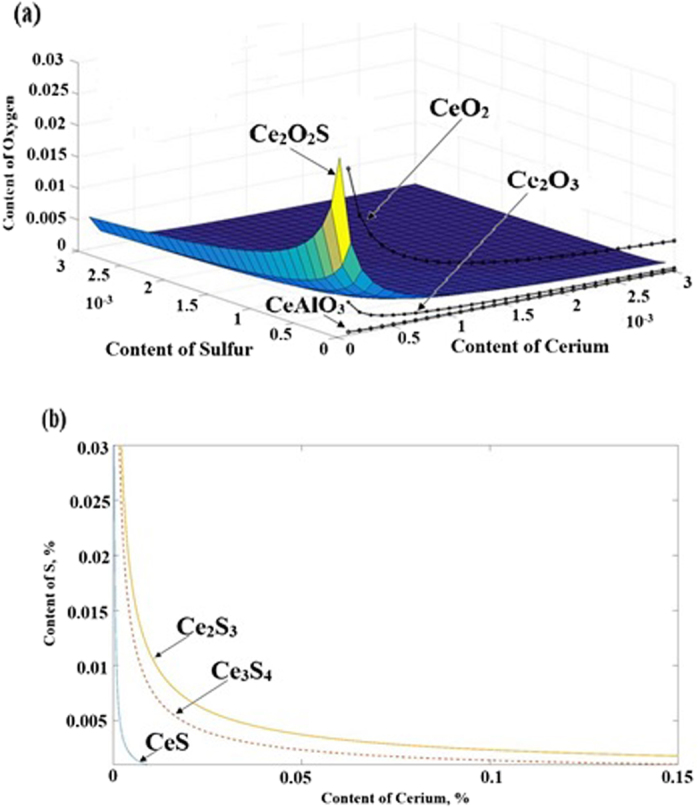
Effects of (**a**) Ce, S and O, (**b**) Ce and S content on the stability of inclusions for sample 0.0235 wt%.

The effects of Ce, S and O contents on the stability of inclusions in SS400 steel were studied, as shown in Fig. 1. According to the results, it can be seen that for a certain amount of cerium, the formation of CeO_2_, Ce_2_O_3_, and CeAlO_3_ needs a higher amount of oxygen, respectively. Besides, for a low amount of cerium, formation of all these inclusions CeO_2_, Ce_2_O_3_, and CeAlO_3_ needs a higher amount of oxygen. For high amount of cerium and S, Ce_2_O_2_S can easily form even in low amount of oxygen. The dominant inclusions are CeO_2_ and Ce_2_O_2_S in this study. Generally speaking, as the binding capacity of RE oxide and oxygen is greater than that of aluminum and oxygen, the liquid steel first produces REAlO_3_, and the reaction equation is [RE] + [Al] + 3[O] = REAlO_3_. Reactions like REAlO_3_ + [RE] + [S] = [Al] + [O] + RE_2_O_2_S & [RE] + [O] = RE_2_O_3_ occur when the addition of RE is increased, which can modify Al_2_O_3_ inclusions, and play a role of desulfurization^22^. RE aluminates, RE oxides, RE sulfur oxide, and RE sulfides will appear in turn by the free energy calculations with RE addition. According to Fig. 1b, it can be seen that for a certain amount of cerium, the formation of Ce_2_S_3_, Ce_3_S_4_ and CeS needs a higher amount of sulfur, respectively. Besides, by increasing the amount of cerium, lower amount of sulfur is needed to form Ce_2_S_3_, Ce_3_S_4_ and CeS.

### Controlling the microstructure, morphology and type of formed Ce-based inclusions

**Figure 2:**
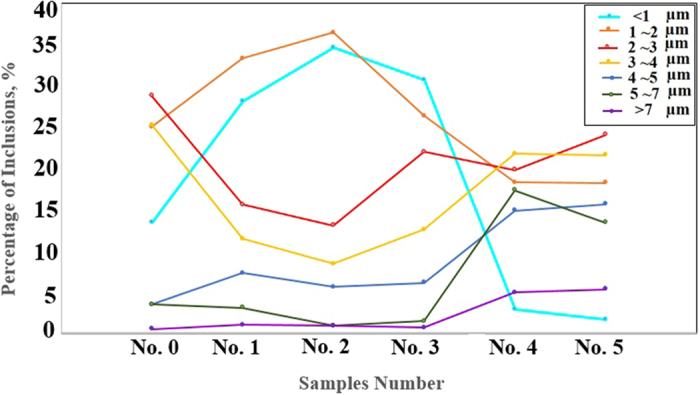
Inclusions size distribution of samples No. 0; No. 1; No. 2; No. 3; No. 4; No. 5.

The inclusions size distribution is studied by ASPEX as shown in Fig. 2. It can be seen that dominant of inclusions in No. 0, are 2~3 μm, the percentage of inclusions in sizes 1~2 μm, and 3~4 μm is nearly 25% and the inclusions >7 μm is only 1%. From sample No. 0 to No. 2, small inclusions are increased while big ones are decreased significantly. The inclusions are fined obviously after treated by Ce for sample No. 2. In sample No. 2, the inclusions <1 & 1~2 μm increase to nearly 33 and 37%, respectively, while the sizes of 2–4 μm inclusions are decreased. However, owing to appreciably increasing of the number density of inclusions, the collision, and aggregation of inclusions will take place when Ce content increases to 0.0235%, so the average size of inclusions in sample No. 4 is bigger than that in sample No. 2 and No. 3. Sample No. 3, big inclusions with the sizes of 2~7 μm are increased but small inclusions <1 & 1~2 μm are decreased. For sample No. 5, with really high amount of cerium, small inclusions are really decreased but big inclusions like inclusions with the size higher than 7 μm are higher for this sample compared with others.

**Figure 3:**
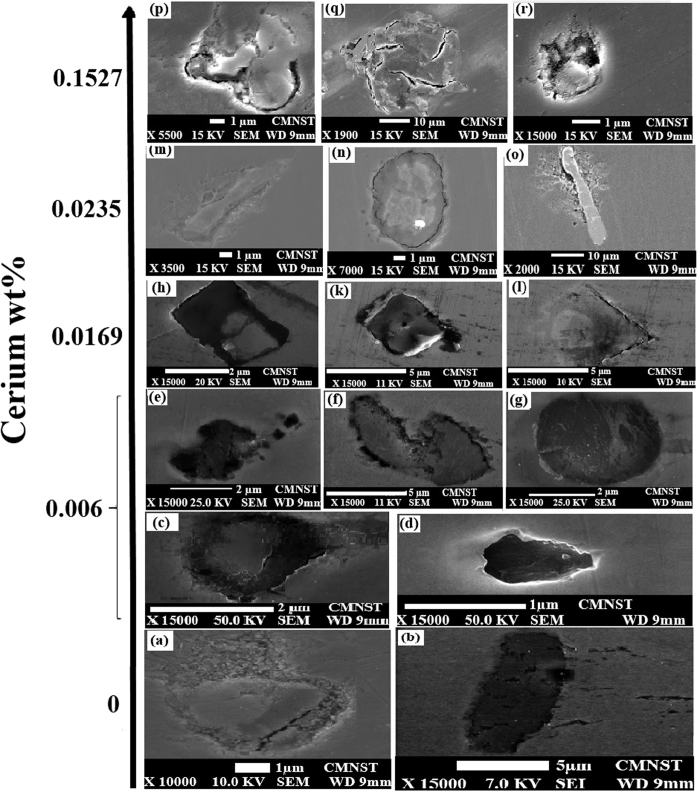
Microstructure and morphology of different samples with changing Ce amount (**a**,**b**) No. 0; (**c**,**d**,**e**,**f** and **g**) No. 2; (**h**,**k**,**l**) No. 3; (**m**,**n**,**o**) No. 4; (**p**,**q**,**r**) No. 5.

Figure 3 show the SEM-EDS microstructure of samples modified with different amount of cerium 0, 0.006, 0.0169, 0.0235 and 0.1527w%, respectively. Fig. 3a, Al_2_O_3_, and Fig. 3b, MnS, are for sample No. 0 (without Ce). Fig. 3c–g are for sample No. 2 (60 ppm Ce). Fig. 3h,k and l are for sample No. 3 (169 ppm Ce). Fig. 3m–o are for sample No. 4 (235 ppm Ce). Figure 3p–r are for sample No. 5 (1527 ppm Ce). Different type of inclusions can be found in these samples. Without adding cerium, MnS is a dominate inclusion in samples, which is presented in Fig. 3b. By adding a small amount of Ce (No. 2, 60 ppm), we will have 5 types of inclusions including complex inclusions without Ce (Fig. 3c), complex inclusions with Ce (Fig. 3d), cerium oxy-sulfide (Fig. 3e), MnS (Fig. 3f), CeAlO_3_ (Fig. 3g). In sample No. 3 (169 ppm Ce), only cerium oxide (Fig. 3k) and cerium oxy-sulfide (Fig. 3h, 3l) inclusions can be found. There is a direct relationship among amount of Ce, S/O ratio and the type of formed inclusions. By increasing the amount of cerium and decreasing S/O, we have a large amount of inclusions most of them cerium oxy-sulfide and cerium oxides, no cerium sulfide has been detected. In contrast, increasing S/O ratio can result in formation of cerium sulfide, which can be found in sample No. 4 (with 235 ppm Ce). In this sample, different inclusions are cerium oxide (Fig. 3m), cerium oxy-sulfide (Fig. 3n) and cerium sulfide (Fig. 3o). Most of inclusions have been cerium oxides or cerium oxy-sulfide, only a small amount of cerium sulfide has been detected, as the S/O ratio is not that much high to form more amount of cerium sulfide. Sample No. 5 with 1527 ppm cerium, has different types of inclusions including cerium oxy-sulfide with a high amount of S (Fig. 3p), cerium oxy-sulfide (Fig. 3q), and small amount of complex inclusion (Fig. 3r).

In order to have a better understanding, the relationship between cerium amount, S/O ratio, and type of formed inclusions as well as the thermodynamic explanations have been summarized in Table 3.

**Figure 4:**
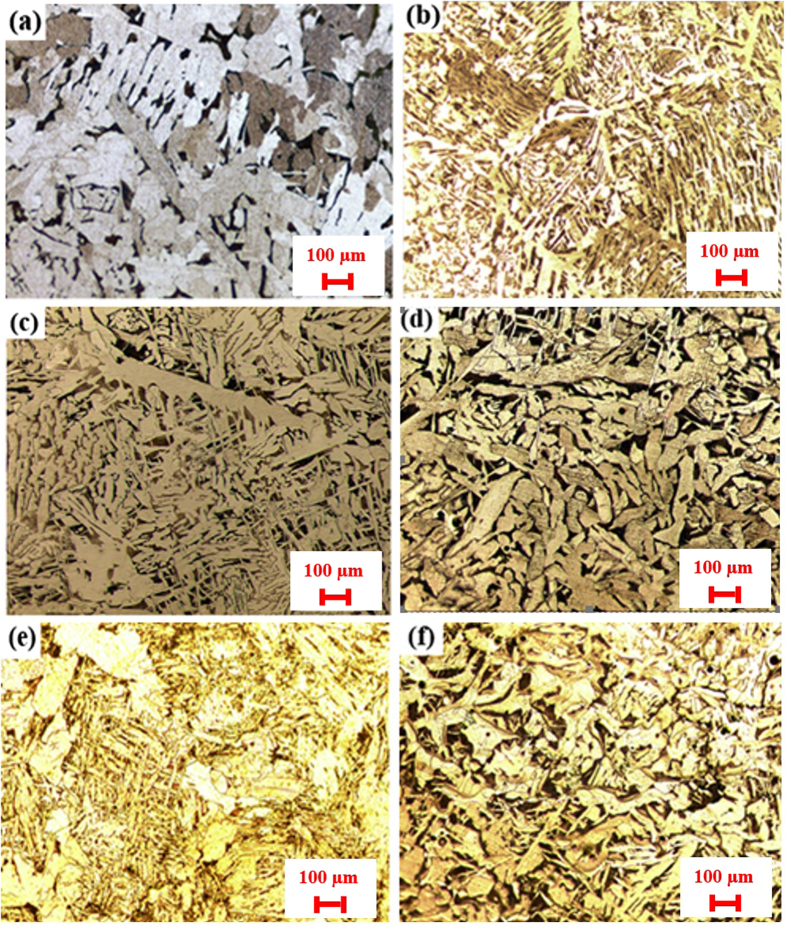
Microstructure and morphology of different samples with changing Ce amount (**a**) No. 0, (**b**) No. 1, (**C**) No. 2, (**d**) No. 3, (**e**) No. 4 and (**f**) No. 5.

Figure 4. show the microstructure of different samples with changing Ce amount after etching by Nital 3% under Optical Microscopy. Figure 4a (No. 0, without Ce) which consist of white plates of polygonal ferrite (PF) and Pearlite (P). Figure 4b (No. 1, 20 ppm Ce) includes aligned side plate ferrite FS(A), Pearlite (P) and small white plates of polygonal ferrite (PF), which are mainly located in grain boundaries. By increasing amount of cerium in sample No. 2 (60 ppm Ce), Fig. 4c, big white plates of polygonal ferrite (PF) and Pearlite (P), PF is becoming elongated, they also located in grain boundaries. Figure 4d is related to sample No. 3 (169 ppm Ce). According to the result, amount of Ce in this sample is lower than sample No. 4 (235 ppm). The number of inclusions are decreased compared with the sample modified with 235 ppm Ce. No AF is found in this sample. Besides, this sample is full of elongated polygonal ferrite and perlite. By increasing amount of Ce to 235 ppm, shown in Fig. 4e, grain refinement and finer microstructure are clearly observed in this result compared with the non-modified sample or even the previous ones. A large amount of acicular ferrite (AF), the white plate shape regions as polygonal ferrite, and also Pearlite can be found in sample No. 4. This sample has 235 ppm which is result in having a reasonable amount of inclusions. Different types of inclusions such as cerium oxides, sulfides and oxy-sulfides are found in this sample as discussed in last section. The amount of cerium is not that much high and is distributed properly. The highest amount of AF is found in this sample compare with other samples. Having cerium sulfide has been effective for the formation of AF. Our results are in agreement with other researchers’^1^,^2^,^3^,^4^,^5^,^6^,^7^,^8^,^9^,^10^,^11^,^12^. Figure 4f is related to sample No. 5 (1527 ppm Ce). Having a high amount of cerium results in increasing the number of inclusions significantly. As a result, high amount of inclusions cannot be effective enough and the inclusions will act like barriers for other. It can be clearly seen that amount of AF is lower than sample No. 4 which can be contributed to this high amount of Ce. A small amount of acicular ferrite (AF), high amount of the white plate shape regions of polygonal ferrite, and also Pearlite can be found. OM results are in agreement with SEM-EDS results. A high amount of inclusions such as cerium oxides, and oxy-sulfides are found in this sample. This sample shows clearly that a proper amount of cerium is necessary; otherwise, the inclusions not only help to grain refinement but also act like barriers to each other.

**Figure 5:**
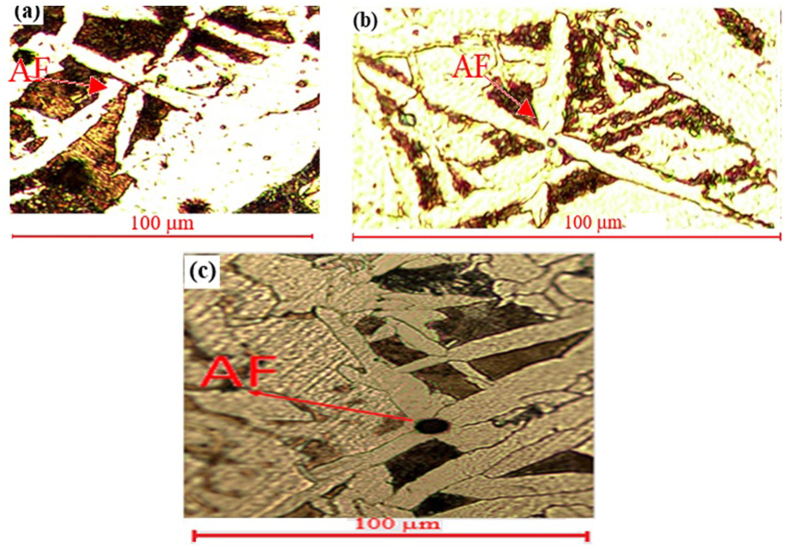
Acicular Ferrite (AF) formed in sample No. 4 after cerium addition.

The formation of IAF in steel was influenced by various factors, such as the composition of inclusions, the amount and size of inclusions, the cooling rate, and the parent austenite grain size (PAGS) and so on^23^,^24^,^25^ pointed out that when the PAGS reached the optimum value, the volume fraction of IAF reached the maximum. Figure 5. shows acicular Ferrite (AF) formed after cerium modification, sample No. 4.

### *In situ* observation of austenite grain refinement by means of confocal microscopy after REM modification

**Figure 6:**
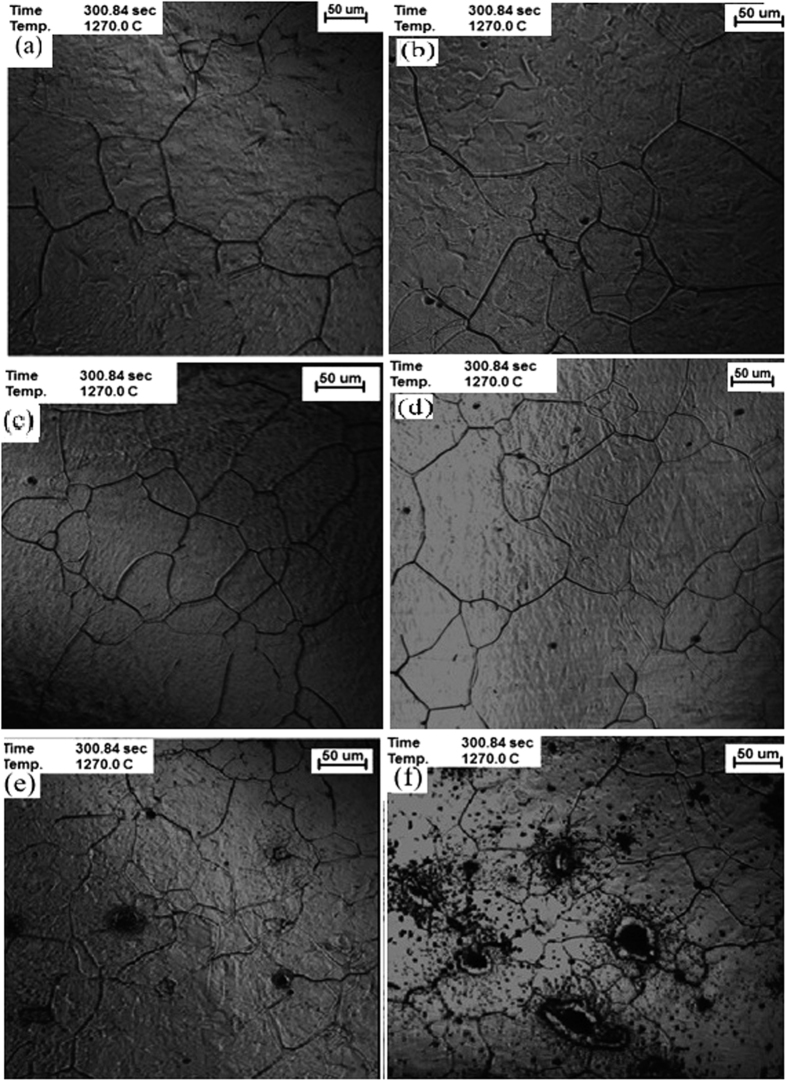
Austenite grain refinement after adding cerium to samples (**a**) No. 0 (No Ce), (**b**) No. 1 (0.002 wt.%), (**C**) No. 2 (0.006 wt.%), (**d**) No. 3 (0.0169 wt.%) No. 4 0.0235 wt.%) and **f**) No. 5 (0.1527 wt.%).

The austenite grain refinement for different samples with different amount of cerium is presented by CM images in Fig. 6. Different samples with different amount of cerium are prepared in order to see which amount of cerium is effective for austenite grain refinement. Besides, the distribution of inclusions will be considered as well. The parent austenite grain size (PAGS) is considered as one of important factors affect the formation of IAF. Bin *et al*.^5^ reported when the PAGS reached the optimum value, the volume fraction of IAF reached the maximum. The amount of cerium is increased from sample No. 0 to No. 5, respectively. According to the results, it can be seen that the austenite grain size is decreased after adding cerium. Austenite grain size for non-modified sample with Ce is about 130 μm. For sample No. 4, a medium amount of inclusions has been distributed and they can probably act as the heterogeneous nucleation site for grains, the average austenite grain size for this sample is around 100 μm. As a result, we will have a finer austenite grain for this sample compare with the others. For sample No. 1 with a small amount of Ce, 20 ppm, only a small refinement can be achieved, but for No. 4 with 235 ppm Ce and No. 5 with higher amount of Ce resulted in austenite grain refinement. Moreover, the amount of inclusions in sample No. 5, is really high. As a result, high amount of cerium cannot be effective enough.

## Conclusion

This study examined the effects of adding cerium on microstructure and morphology of Ce-based inclusions formed in commercial SS400. The results are summarized as follows:

1. Different samples with different amount of cerium have been prepared. For sample with 0.0235 wt.% cerium and S/O ratio around 7, the type of inclusions will be large amount of cerium oxide, cerium oxy-sulfide, and small amount of cerium sulfide.
2. As the binding capacity of Ce oxide and oxygen is greater than that of aluminum and oxygen, the liquid steel first produces CeAlO_3_, and the reaction equation is [Ce] + [Al] + 3[O] = CeAlO_3_. Reactions like CeAlO3 + [Ce] + [S] = [Al] + [O] + Ce_2_O_2_S & [Ce] + [O] = Ce_2_O_3_ will occur when the addition of Ce is increased, which can modify Al_2_O_3_ inclusions, and play a role of desulfurization. Ce aluminates, Ce oxides, Ce sulfur oxide, and Ce sulfides will appear in turn by the free energy calculations with Ce addition.
3. By increasing the amount of cerium and decreasing S/O, a large amount of inclusions can be detected which most of them are cerium oxy-sulfide. It’s worth to mention no cerium μm sulfide is detected when S/O ratio is low. There is a direct relationship among amount of Ce, S/O ratio and the type of formed inclusions. Besides, having a high amount of cerium results in increasing the number of inclusions significantly. As a result, high amount of inclusions cannot be effective enough and the inclusions will act like barriers for others.
4. Inclusions with a size of about 4∼7 μm can serve as heterogeneous nucleation sites for IAF formation. The amount of cerium is not that much high and is distributed properly. The highest amount of AF is found in the sample with 0.0235w% Ce compare with other samples. Having cerium sulfide has been effective for the formation of IAF.

## Additional Information

**How to cite this article:** Adabavazeh, Z. *et al*. Effect of Adding Cerium on Microstructure And Morphology of Ce-Based Inclusions Formed in Low-Carbon Steel. *Sci. Rep.* **7**, 46503; doi: 10.1038/srep46503 (2017).

**Publisher's note:** Springer Nature remains neutral with regard to jurisdictional claims in published maps and institutional affiliations.

## Figures and Tables

**Table 1:** Chemical composition of the investigated steel SS400 (Weight Percent).

Sample	C	Mn	Si	Al	P	S	O	Ce	S/O
No. 5	0.205	1.397	0.403	0.214	0.0110	0.0075	0.0080	0.1527	<1
No. 4	0.201	1.300	0.400	0.200	0.0060	0.0049	0.0007	0.0235	7.3
No. 3	0.185	1.508	0.373	0.183	0.0110	0.0007	0.0060	0.0169	<1
No. 2	0.196	1.341	0.382	0.214	0.0080	0.0019	0.0017	0.0060	∼1
No. 1	0.181	1.343	0.401	0.186	0.0080	0.0003	0.0012	0.0020	<1
No. 0	0.094	1.342	0.390	0.108	0.0070	0.0017	0.0037	0	<1

**Table 2:** Interaction coefficient 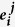 of various elements in liquid steel at 1873K.

Element (*i, j*)	C	N	O	Si	Mn	P	S	Al	Ce
O	−0.45	0.057	−0.2	−0.131	−0.021	0.07	−0.133	−3.9	−0.57
Ce	0.397	−6.612	−5.03	0	0	1.77	−10.34	−2.67	−0.008
S	0.11	0.01	−0.27	0.063	−0.026	0.029	−0.028	0.035	−2.36
Al	0.091	−0.058	−6.6	0.0056	0	0	0.03	0.045	−0.5114

**Table 3:** Summary of the relationship between amount of Ce, S/O ratio and type of formed inclusions.

Sample	cerium[wt.%]	Expected inclusions, procedure of formation of inclusions
No. 0	0	**SEM Results: MnS, Al**_**2**_**O**_**3**_
		EXPLANATIONS: Most of inclusions are MnS, Al_2_O_3_ can also be found. As we did not add cerium, Gibbs free energy for formation of MnS is favorable.
No. 2	0.006	**SEM Results: cerium oxy-sulfide, MnS, CeAlO**_**3**_**, Complex with cerium, Complex without Ce**
		EXPLANATIONS: As the binding capacity of RE and oxygen is greater than that of aluminum and oxygen, the liquid steel first produces REAlO3, and the reaction equation is: [RE] + [Al] + 3[O] = REAlO_3_ At the same time some of REAlO_3_ can react with RE and S: REAlO_3_ + [RE] + [S] = [Al] + [O] + RE_2_O_2_S
No. 3	0.0169	**SEM Results: cerium oxide, cerium oxy-sulfide**
		EXPLANATIONS: Reactions like REAlO_3_ + [RE] + [S] = [Al] + [O] + RE_2_O_2_S & [RE] + [O] = RE_2_O_3_ will occur when the addition of RE is increased, which can modify Al_2_O_3_, and play a role of desulfurization.
No. 4	0.0235	**SEM Results: cerium oxide, cerium oxy-sulfide, small amount of cerium sulfide**
		EXPLANATIONS: RE aluminates, RE oxides, RE sulfur oxide, and RE sulfides will appear in turn by the free energy calculations with RE addition. The amount of Ce is high enough as a result REAlO_3_ is transformed to RE_2_O_2_S. As a result, no REAlO_3_ is detected.
No. 5	0.1527	**SEM Results: A large amount of inclusions most of them cerium oxy-sulfide, no cerium sulfide**
		EXPLANATIONS: Amount of S is not high enough to form cerium sulfide as a result no CeS.
